# Epicardial Adipose Tissue as an Independent Cardiometabolic Risk Factor for Coronary Artery Disease

**DOI:** 10.7759/cureus.25578

**Published:** 2022-06-01

**Authors:** Nikoleta Karampetsou, Leonidas Alexopoulos, Aggeliki Minia, Vaia Pliaka, Nikos Tsolakos, Konstantinos Kontzoglou, Despoina N Perrea, Paulos Patapis

**Affiliations:** 1 Experimental Surgery and Surgical Research, National and Kapodistrian University of Athens, Athens, GRC; 2 Mechanical Engineering, National Technical University of Athens, Athens, GRC; 3 Biotechnology, ProtATonce Ltd., Athens, GRC; 4 Propaedeutic Surgery, National and Kapodistrian University of Athens, Athens, GRC; 5 Surgery, National and Kapodistrian University of Athens, Athens, GRC

**Keywords:** visceral adiposity, inflammation, cad: coronary artery disease, coronary artery atherosclerosis, epicardial adipose tissue

## Abstract

During the last decades, visceral adiposity has been at the forefront of scientific research because of its complex role in the pathogenesis of cardiovascular diseases. Epicardial adipose tissue (EAT) is the visceral lipid compartment between the myocardium and the visceral pericardium. Due to their unobstructed anatomic vicinity, epicardial fat and myocardium are nourished by the same microcirculation. It is widely known that EAT serves as an energy lipid source and thermoregulator for the human heart. In addition to this, epicardial fat exerts highly protective effects since it releases a great variety of anti-inflammatory molecules to the adjacent cardiac muscle. Taking into account the unique properties of human EAT, it is undoubtedly a key factor in cardiac physiology since it facilitates complex heart functions. Under pathological circumstances, however, epicardial fat promotes coronary atherosclerosis in a variety of ways. Therefore, the accurate estimation of epicardial fat thickness and volume could be utilized as an early detecting method and future medication target for coronary artery disease (CAD) elimination. Throughout the years, several therapeutic approaches for dysfunctional human EAT have been proposed. A balanced healthy diet, aerobic and anaerobic physical activity, bariatric surgery, and pharmacological treatment with either traditional or novel antidiabetic and antilipidemic drugs are some of the established medical approaches. In the present article, we review the current knowledge regarding the anatomic and physiological characteristics of epicardial fat. In addition to this, we describe the pathogenic mechanisms which refer to the crosstalk between epicardial fat alteration and coronary arterial atherosclerosis development. Lastly, we present both lifestyle and pharmacological methods as possible treatment options for EAT dysfunction.

## Introduction and background

Coronary artery disease (CAD) is a considerable health condition affecting millions of individuals all over the world [1]. According to the American Heart Association, 840,768 individuals died due to coronary artery disorder in the United States of America (USA) in 2016 [2]. Recent data suggest that CAD remains the principal cause of human mortality and morbidity in both developed and developing countries [3]. Stable angina, acute coronary syndromes (myocardial infarction, unstable angina), and sudden cardiac death are some of CAD’s clinical manifestations [3]. Coronary atherosclerosis, characterized by remodeling and occlusion in the coronary arterial trees, is proposed to be the main etiopathogenic mechanism of CAD [4]. To date, numerous risk factors either modifiable (e.g., arterial hypertension, dyslipidemia, smoking, obesity) or not (e.g., family history of acute coronary syndrome in relatively early adulthood, sex, age) have been associated with the uprising incidence of CAD [5].

In recent years, adipose tissue has been at the center of scientific medical research [6]. A growing body of data has indicated different and crucial roles of human adiposity, from energy fuel to thermoregulation and injury protection [6,7]. In addition to this, data from prestigious experimental and clinical studies have recognized both the endocrine and paracrine effects of adipose tissue on animals and humans [7]. It is now well-established that excessive adiposity releases plenty of inflammatory cytokines leading to a low-grade inflammatory microenvironment, endothelial dysfunction, increase in oxidative stress, and therefore, coronary atherosclerosis [8].

Epicardial adipose tissue (EAT) is the visceral fat unit localized between the myocardium and the inner pericardium, surrounding major coronary vessels [9]. Due to their close anatomic relationship, epicardial fat and the proximal myocardium share the same vessel microcirculation [9]. Numerous clinical and basic research studies have been conducted during the last two decades in order to investigate epicardial fat unique features [10]. To our knowledge, epicardial fat is a biologically active endocrine organ with several paracrine and vasocrine effects on the proximal myocardium [11]. Nowadays, it is accepted that epicardial fat plays a significant and cardioprotective role in normal heart function [8]. However, recent data suggest that abnormal epicardial fat could lead to adverse cardiovascular outcomes through several etiopathogenic mechanisms [12]. In fact, several clinical studies have demonstrated a direct correlation between CAD and dysfunctional EAT [13]. In this way, the unique transcriptome of epicardial fat has become a subject of thorough scientific investigation [9].

In this paper, we briefly report the anatomy, physiology, and quantification methods of EAT. Also discussed are the pathophysiology mechanisms in which abnormal epicardial fat induces CAD. Lastly, we present potential therapeutic approaches in order to modify abnormal EAT's function in CAD. 

## Review

Anatomy

Epicardial fat is the adipose tissue localized between myocardium and the epicardium (the visceral surface of pericardium), covering approximately 80% of the total heart mass [14]. It is mainly detected in the interventricular and atrioventricular chambers, the apex of the heart and the free wall of the right ventricle surrounding major branches of coronary arterial tree [14]. It is distinguished in myocardial EAT (the adipose tissue just over the myocardial surface) and pericoronary EAT (the fat depot directly located around the coronary arteries) [15]. Epicardial and paracardial fat depots compose pericardial adipose tissue (PAT) [16]. To our knowledge, PAT and epicardial fat share similar morphological features [15]. However, they present different embryological origin, since PAT derives from thoracic mesoderm and EAT from splanchnopleuric mesoderm [16]. Furthermore, EAT is supplied with oxygen and other nutritious ingredients by small branches of coronary vessels, whereas PAT is nourished by thoracic arteries [16]. Histologically, EAT consists of various types of cells, such as adipocytes, inflammatory and immune cells, nerve cells and vascular ones [17]. Additionally, there is no intervening anatomic barrier between myocardium and epicardial fat [18]. As a result, the same small vessel circulation is shared by both of them [18].

Several factors, including age, race, body mass index (BMI) and sex have been associated to anatomical alteration of EAT distribution in the human heart [10]. It has been described that by the age of 65 years EAT thickness increases by almost 22% [19]. Caucasian men are also characterized by greater epicardial fat thickness than black men [20]. Obesity is a triggering factor of increase in visceral adiposity [10]. Thus, abnormal BMI is also correlated to higher EAT mass [10]. Obese women seem to present greater EAT mass than men [10].

Physiology

EAT as an Energy Fat Source

**Figure 1:**
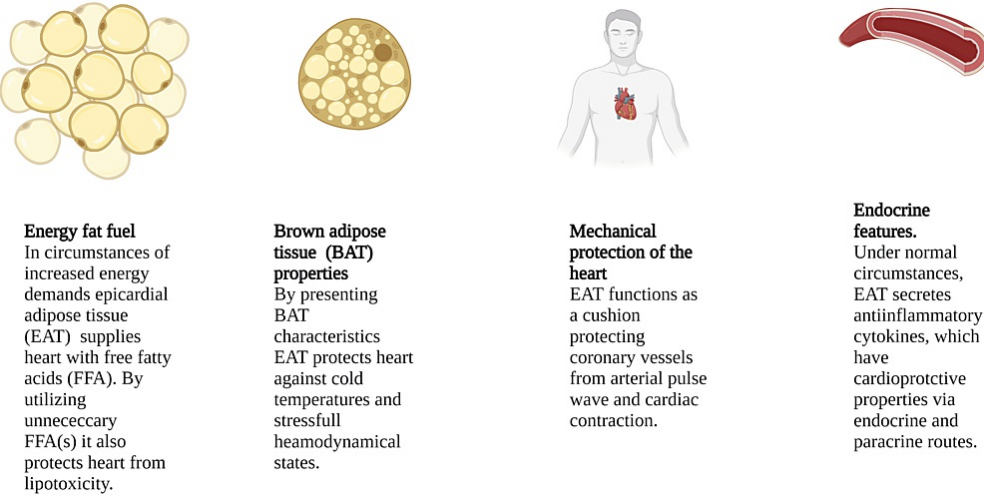
Physiological functions of epicardial adipose tissue (EAT) EAT: Epicardial adipose tissue, FFA: Free fatty acid, BAT: Brown adipose tissue This figure was created by the author of the article, Nikoleta Karampetsou.

Numerous investigators have attempted to identify the physiological functions of epicardial fat (Figure 1) [9]. Firstly, EAT is a vital energy lipid storage for myocardium in periods of elevated energy requirements [21]. It has been demonstrated that epicardial fat uptakes and releases free fatty acids (FFA) to a much greater degree than other visceral adipose tissues [22]. Free fatty acids are the dominant energy source for a healthy heart’s contraction, whereas their oxidation covers up to 70% of the heart’s total energy requirements [22]. Impressively, fatty acid-binding protein 4 (FABP4) is extensively expressed by human epicardial adipocytes [23]. This protein transfers free fatty acids from EAT to the adjacent heart muscle via either vasocrine or paracrine routes [23]. Moreover, epicardial fat quickly utilizes redundant free fatty acids since it converts them into lipid storage units for future energy myocardial demands. [14]. Thus, myocardium may be efficiently protected from lipotoxicity [11].

Mechanical Protection

Due to its location in the atrioventricular and interventricular chambers and its vicinity to coronary vessels, EAT serves as a cushion [24]. In this way, it protects coronary arteries from violent distortion triggered by arterial pulse wave and myocardial contraction [24]. Furthermore, it acts as a supplemental anatomic layer for the total heart mass in case of mechanical injury [24].

Brown Adipose Tissue Properties

Brown adipose tissue (BAT) is a subject of vigorous scientific investigation since its role in human beings has not yet been clarified [25]. Protection against low temperatures via thermoregulatory mechanisms and stressful hemodynamic states, such as ischemia and hypoxia are the main functions of brown fat [25]. It has been described that in neonates epicardial brown fat cells are the majority of EAT adipocytes [26]. However, from infant to adult life the number of brown adipocytes annually decreases [26]. Recently, it has been proposed that EAT could be defined as beige adipose tissue, since brown fat cells are gradually replaced by small unilocular white adipocytes, similar to beige lineage ones [25]. Uncoupling protein-1 (UCP-1) and other brown specific proteins are impressively greatly expressed in human EAT [27]. Interestingly, UCP-1 presents higher concentration in human EAT than in other visceral fat units [27]. Brown specific fat proteins facilitate heart thermoregulation and protect it against stressful conditions, as mentioned above [25]. According to Sacks et al., advanced coronary atherosclerosis is associated with the elimination of these proteins in favor of significant elevation of inflammatory ones [28]. 

Epicardial Adipose Tissue as an Endocrine Organ

Epicardial adipose tissue is not only an energy fat source but also an active endocrine organ with several vasocrine and paracrine effects [29]. Under physiological circumstances, epicardial fat secretes plenty of beneficial cytokines, such as adiponectin, adrenomedullin and omentin-1 which present anti-atherogenic, anti-inflammatory and antithrombotic properties [29]. Paracrine and vasocrine pathways have been suggested as the two main interaction routes between EAT and the human heart [13]. The paracrine method promotes the adipokines’ diffusion from the pericoronary adipose tissue to the arterial wall and the direct interaction with the endothelial and vascular smooth cells [30]. The vasocrine way includes the direct transit of free fatty acids and adipokines into the coronary lumen's vasa vasorum [31]. The adipocytokines mentioned above activate adenosine monophosphate-activated protein kinase (AMPK), an enzyme mostly produced by heart and skeletal muscles [32, 33]. Under cellular stress states and low energy supplies, AMPK induces catabolic pathways and restricts anabolic ones in order to increase cellular adenosine triphosphate (ATP) levels and regulates glucose and free fatty acid uptake [32,33].

Adiponectin is a protein hormone mainly produced by adipocytes. Epicardial fat expresses adiponectin receptor 1 (AdipoR1) and adiponectin receptor 2 (AdipoR2) [34]. Adiponectin connection to these receptors leads to free fatty oxidation, triacylglycerol (TAG) turnover, and a decrease in fat storage in human EAT [35]. Secondly, through AMPK activation adiponectin restricts the production of acetyl coenzyme A (acetyl-CoA) [35]. In this way, both glucose levels and fatty oxidation are regulated [35]. Noticeably, adiponectin also induces an anti-inflammatory microenvironment in the human heart since it inhibits the production of inflammatory cytokines, such as tumor necrosis factor-alpha (TNF-α) and interleukin-6 (IL-6) [36].

Adrenomedullin is a protein which presents crucial cardioprotective features. It interacts with the calcitonin receptor-like receptor (CRLR) and stimulates protein kinase A (PKA) in cardiac muscle cells [37]. Therefore, calcium channels are activated and calcium is released in cardiomyocytes cytoplasm leading to the elevation of cardiac output [38]. Except for this function, it has been reported that adrenomedullin inhibits endothelial cell apoptosis and reduces oxidative stress [38]. Another adipocytokine that is highly expressed in EAT under normal circumstances is omentin-1 [36]. It is characterized by anti-atherogenic properties since it induces the activation of anti-inflammatory M2 macrophages and subdues foam cell formation linked to oxidization of low-density lipoprotein (LDL) [39]. In their study, Du et al. demonstrated that omentin-1 expressed in EAT was significantly reduced in patients with CAD than in individuals without [36]. Finally, it is now evident that EAT in comparison to subcutaneous adipose tissue (SAT) releases greater levels of proteins associated to potassium channel interaction, biosynthesis and wound healing [40].

Epicardial adipose tissue: Assessment techniques

Nowadays, growing evidence suggests that excessive epicardial adiposity is an independent risk factor for cardiovascular disease development [41]. Increased EAT thickness and volume are clinical markers of excessive visceral fat accumulation [41]. Thus, accurate quantification of EAT could serve both as a crucial prognostic tool and as a medication target for cardiovascular diseases, especially CAD [41]. In 2003, Iacobellis et al. first assessed EAT thickness through two-dimensional echocardiography [42]. Epicardial fat appears as an echo-free space above the right ventricle’s free wall, where it is more prominent [42]. Parasternal long-axis ultrasound images in combination with short-axis ones at the end of systole during three cardiac cycles are performed in order to achieve precise measurement of EAT [42]. To our knowledge, 5 mm of epicardial fat thickness recorded in transthoracic echocardiography has been proposed as a cut-off value and prognostic risk factor for CAD [10]. Transthoracic echocardiography remains an easy, economic, noninvasive and easily reproduced procedure of EAT quantification [11]. However, total eat volume cannot be estimated by 2D-echo [11]. Additionally, the quality of measurements highly depends on the operator’s experience [11, 42].

Cardiac magnetic resonance imaging ( MRI) and multislice computed tomography (MCT) are novel and promising imaging techniques for reliable volumetric assessment of epicardial fat [31]. The MRI provides explicit information regarding total EAT volume and mass by using the spin-echo sequence technique and without radiation exposure to patients [43]. Specifically, manual contouring of epicardial fat region in short-axis views at the end of diastole during several cardiac cycles provides precise quantification of EAT volume in each slice [31]. The total of the above measurements indicates the total EAT volume in the human heart [31]. Despite the advantages, MRI is still an expensive and hardly accessible method in daily clinical practice [44].

On the other hand, either contrast or non-contrast enhanced MCT is the most desirable EAT imaging method, since it has better spatial resolution and not only identifies exact total EAT volume but also evaluates coronary arterial calcification [31,45]. In addition to this, cardiac computed tomography angiography (CCTA) is a similar method which not only estimates coronary artery stenosis but also recognizes dangerous atherosclerotic plaque characteristics, such as vulnerable atheromas [46]. Consequently, it may be suggested as a valuable risk stratification tool [46]. However, the cost, the restricted availability and the remarkable exposure to ionizing radiation are major disadvantages of both MCT and CCTA [45, 47].

Despite its several limitations, two-dimensional echocardiography remains the most preferable and cost-effective method of epicardial fat evaluation in daily clinical practice (Table 1).

**Table 1:** Pros and cons of main current imaging methods for epicardial adipose tissue (EAT) quantification EAT: Epicardial adipose tissue, MRI: Magnetic resonance imaging, CT: Computed tomography

Imaging techniques	Availability	Cost	Radiation	Epicardial adipose tissue (EAT) thickness assessment	EAT volume assessment	Coronary artery calcification
Echocardiography	easily available	low	no	yes	no	no
Magnetic resonance imaging (MRI)	not easily available	very high	no	yes	yes	no
Computed tomography (CT)	not easily available	high	yes	yes	yes	yes

Epicardial adipose tissue and coronary artery disease

Excessive adiposity has a well-established role in coronary atherosclerosis by both secreting plenty of active cytokines and regulating insulin sensitivity [48]. During the last two decades, interest regarding EAT and CAD has been growing rapidly [12]. Unobstructed anatomical proximity of EAT and coronary arterial tree indicates the substantial role of it in coronary atherosclerosis progression [8]. In addition to local vicinity, however, EAT seems to induce CAD in numerous ways [8].

Coronary artery disease is inflammatory in nature [3]. Inflammatory properties of epicardial fat have been investigated since the early 2000s [11]. Multiple studies have reported dense infiltration of inflammatory cells in human EAT [11]. In 2011, Hirata et al. demonstrated a greater concentration of inflammatory M1 macrophages than anti-inflammatory M2 macrophages in EAT samples derived from patients suffering from advanced CAD [49]. It is now well-known that the increased prevalence of M1 macrophages persuades coronary atheromas instability and thereafter rupture [49]. Except for macrophages, the presence of mast cells, B lymphocytes, T lymphocytes, and dendritic cells in EAT has also been associated with coronary atherosclerosis [50]. All the previously mentioned cells express in their membranes Toll-like receptors (TLRs) [51]. Extracellular ligands, such as saturated fatty acids connect to TLRs and promote immune response [51]. Particularly, nuclear factor-κB (NF-κΒ) and JUNN-terminal kinase (JNK) are activated and induce the upregulation of inflammatory molecules in EAT [51]. In their study, Baker et al. demonstrated elevated activation of NF-κB and JNK pathophysiology pathways in EAT biopsies of people suffering from advanced CAD [51].

In a benchmark study, Mazurek et al. observed elevated concentrations of inflammatory mediators in human EAT [15]. Interleukin-6 (IL-6), interleukin-1 (IL-1), and TNF-a were all upregulated in subjects suffering from CAD [15]. In addition to this, the inflammation level was higher in epicardial fat than subcutaneous adipose tissue in these subjects [15]. High TNF-α concentrations increase IL-6 levels [52]. Both of them elevate endothelial and vascular smooth cell permeability, while they also have a negative influence on insulin tissue sensitivity [52]. Since then, several studies have identified genes encoding other inflammatory adipokines, such as resistin and chemerin [36,52]. Due to the lack of anatomic intervening barrier, inflammatory cytokines expressed by EAT affect coronary arteries through paracrine and vasocrine pathways [53]. Lately, it has been shown that elevated abnormal EAT proteasome leads to thicker EAT layer, more intense chronic inflammation and therefore more severe CAD [15,44,54]. By contrast, cardioprotective cytokines, such as adiponectin and adrenomedullin were found extremely low in EAT samples from people with CAD [11]. The reduction of such anti-inflammatory molecules leads to further coronary inflammation [11]. Finally, leptin is another highly expressed hormone in the pericoronary EAT [55]. Leptin expression is upregulated by IL-6 and downregulated by TNF-a [56]. Increased leptin levels promote endothelial cell permeability and induce monocytes and macrophages adhesion, and destabilize atheromatous plaques in the coronary lumen [55]. It has been noticed that the adiponectin/leptin ratio in EAT decreases under pathological conditions and is a novel risk factor for CAD [55,57].

Epicardial adipose tissue has also been gaining interest regarding oxidative stress [53]. Recently, it has been proposed that epicardial fat in humans suffering from CAD presents higher concentration of reactive oxygen species (ROS) [58]. In their study, Demir et al. found increased total oxidative stress (TOS) in epicardial fat compartment of patients suffering from CAD, and metabolic syndrome [59]. The ROS activates various transcription factors which result in messenger ribonucleic acid (mRNA) encoding of genes involved in local inflammation [59]. Cellular stress mediators, such as mitogen-activated protein kinase kinase kinase 5 (MAP3K5) are highly expressed by epicardial adipocytes leading to cellular apoptosis and further endothelial dysfunction [40]. In this way, the imbalance between ROS and inflammatory factors results in chronic inflammation, endothelial dysfunction, and consequently, coronary atherosclerosis. [40]

Lipotoxicity is deemed to be another pathophysiology mechanism which promotes EAT-induced coronary atherosclerosis [60]. Nowadays, it is known that excessive epicardial fat provokes imbalance in lipid and glucose metabolism [60]. Redundant epicardial fat secretes a high amount of free fatty acids, which are then accumulated in coronary artery lumen promoting atheromatous plaque development [10,13]. Remarkably, phospholipase A2 and endothelial lipase are significantly elevated in EAT of individuals with CAD [60]. These proteins participate in lipid metabolism pathways and could serve in further ectopic fat accumulation in adjacent coronary vessels [60]. Furthermore, epicardial fat is deemed to be an insulin-resistant lipid compartment [61]. In fact, it is characterized by lower glucose utilization rate than other visceral fat tissues [61]. Glucose-transporter 4 (GLUT4) is an intracellular protein mainly expressed in muscle cells and adipocytes [62]. It regulates glucose uptake in fat and muscle cells after insulin cascade activation [62]. Epicardial adipocytes are generally characterized by lower levels of glucose transporter type 4 (GLUT-4) [32]. However, in diabetic individuals also suffering from coronary atherosclerosis, GLUT4 levels in EAT were even lower, underlying in this way the local role of insulin-induced atherogenesis in CAD [62].

All these pathophysiology pathways underlie a direct relationship between epicardial fat and CAD [9]. Consequently, EAT may be used as a screening and risk stratification tool [48]. Both EAT thickness and volume have been measured higher in humans with CAD than in those without [13]. In their clinical investigation, Fahri et al. correlated elevated mean EAT thickness with critical atheromatosis as demonstrated by Syntax and Gensini scores [63]. Increased coronary artery calcium score (CACS >10) was also observed in patients with high EAT volume as measured by either CT or MRI [64]. Recently, Cosson et al. proposed the quantification of EAT volume as an independent parameter in the CACS determination in diabetic patients [65]. In another study, elevated EAT volume was tightly associated with vulnerable atheromatic plaques in patients suffering from symptomatic atherosclerosis but had zero CACS [66]. Taking this into consideration, the EAT assessment may be used for early diagnosis of non-calcified and unstable atheromatous plaques [66]. Moreover, epicardial fat is not equally distributed in the whole heart mass [57]. Because of their anatomic proximity, coronary arteries are mainly affected by pericoronary EAT [64]. A higher pericoronary EAT volume has been referred as a determining risk factor leading to more severe CAD and calcification in postmenopausal women [67]. Same results were also observed in prediabetic patients with history of myocardial infraction [57]. Lastly, the increased levels of inflammatory cytokines in serum and EAT samples of humans with CAD underlie the crucial role of abnormal epicardial fat proteasome in coronary atherosclerosis progression. [18,29,30]

Therapeutic targets in epicardial adipose tissue

Lifestyle Modifications

**Figure 2:**
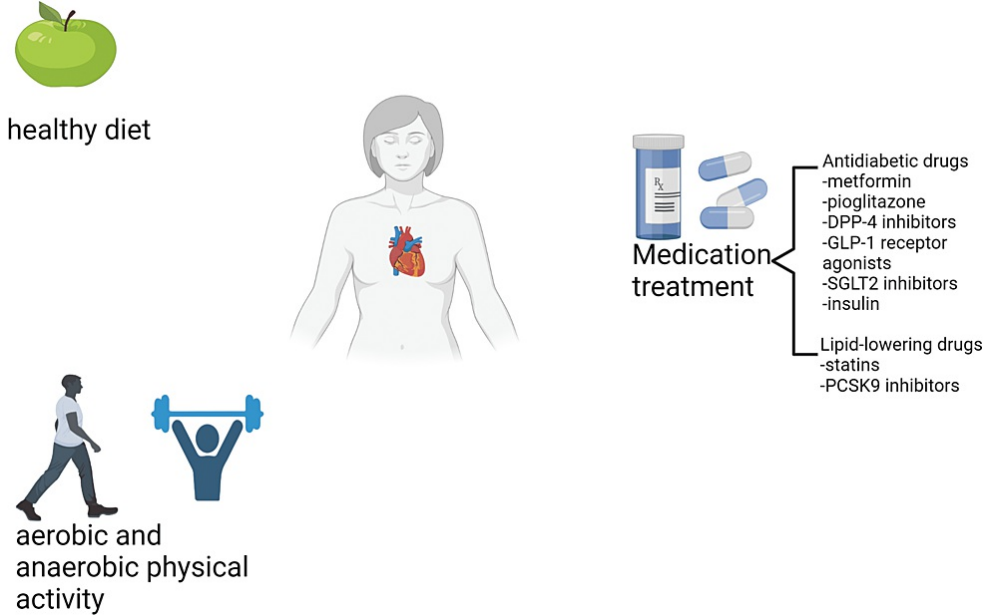
Potential medical approaches for abnormal epicardial fat DPP-4 inhibitors: Dipeptidyl peptidase-4 inhibitors, GLP-1 receptor agonists: Glucagon-like Peptide-1 receptor agonists, SGLT2 inhibitors: Sodium-glucose cotransporter-2 inhibitors, PCSK9 inhibitors: Proprotein convertase subtilisin/kexin type 9 inhibitors This figure was created by the author, Nikoleta Karampetsou.

Undoubtedly, epicardial fat is a novel cardiovascular risk factor [29]. Despite the vigorous investigation of the main pathogenic mechanisms of EAT, there is still little knowledge regarding suitable medical treatment of this pathological epicardial fat compartment (Figure 2) [29]. Lifestyle modifications, such as weight loss, aerobic and anaerobic physical exercise seem to be key factors in achieving EAT thickness reduction [11]. Iacobellis et al. noticed a significant decrease in EAT thickness of critically obese patients (BMI 45± 5 kg/m²) who attended a low-calorie diet for up to six months [68]. In a recent pilot study, Konwerski et al. investigated the effect of intense aerobic physical exercise on epicardial fat volume and metabolic profile among 30 ultramarathon amateur athletes and eight individuals with a sedentary lifestyle (control group) [69]. The EAT volume as measured by cardiac MRI was significantly lower in ultramarathon athletes than in the control group [69]. Furthermore, ultra-runners also presented better metabolic profile (lower lipid concentration and lower BMI) as well as lower levels of plasma IL-6 when compared to the control group [69]. In a recent meta-analysis of 10 studies, similar positive effects of endurance training on EAT were also noticed [70]. Additionally, it is widely known that obesity is associated with coronary atherosclerosis and excessive visceral adiposity, including increase in total EAT mass [71]. In 2018, Altin et al. investigated the potential role of bariatric surgery, and specifically sleeve gastrectomy, in epicardial fat thickness. They reported the eliminated EAT thickness in all echocardiography measurements after bariatric surgery [71].

Medication Treatment

With the exception of lifestyle modifications, several medications have been under investigation in order to indicate their probable role in abnormal EAT alteration [72]. Antilipidemic drugs such as statins not only reduce EAT thickness but also restrict pathological metabolic activity of epicardial fat due to their pleiotropic properties [72]. Firstly, statins inhibit competitively the transformation of hydroxymethyl glutaryl-CoA to mevalonic acid, resulting in increased expression of LDL receptors. Furthermore, statins present anti-inflammatory features since they restrict the growth of macrophages, reduce immune cell adhesion in atheromatous plaques and suppress proinflammatory cytokines secretion [73]. Raggi et al. formed a randomized controlled trial for one year with 420 postmenopausal women, in which 194 were treated with 80 mg atorvastatin whereas the other 226 received 40 mg pravastatin. All the patients underwent chest computed tomography in order to quantify epicardial fat attenuation in Hounsfield units (EAT HUs). The EAT HUs were measured in the area of right coronary artery. Atorvastatin reduced EAT volume more efficiently than pravastatin. However, both of them decreased EAT HUs almost similarly, demonstrating the anti-inflammatory properties of statins [74]. Proprotein convertase subtilisin/kexin type 9 (PSCK9) is an inflammatory mediator highly produced by visceral fat depots, especially EAT [75]. It promotes arterial atherosclerosis through direct interaction with LDL receptors and monocytes adhesion in the atheromatous plaque [75]. The PCSK9 inhibitors are lipid-lowering drugs which may be involved in the reduction of EAT thickness [76]. In 2020, Galvez et al. observed that in 24 patients who received either evolocumab or alirocumab for six months, EAT thickness was significantly reduced as measured by transthoracic echocardiography [76].

To the best of our knowledge, several medications used for diabetes and/or obesity may also provide cardioprotective benefits beyond glycaemic control [77]. Metformin remains the most commonly administrated hypoglycemic substance worldwide [78]. It is proposed as the gold-standard for initial therapy of type-2 diabetes [78]. Several studies have demonstrated its determining role in blood glucose reduction and increase in insulin sensitivity in target organs, such as visceral fat [57]. In 2019, Ziyrek et al. reported that the initiation of metformin in 40 diabetic subjects resulted in significant decrease in EAT thickness three months after [78]. Pioglitazone belongs to a family of hypoglycemic drugs called thiazolidinediones [79]. It stimulates both peroxisome proliferator-activated receptor gamma (PPAR-γ) and proliferator-activated receptor alpha (PPAR-α) [79]. Thus, it inhibits the secretion of pro-inflammatory cytokines in visceral fat depots, alternating the metabolic profile [28]. Grosso et al. reported important depletion of cytokines( IL-6, TNF-a, resistin, etc.) expressed in EAT biopsies derived from patients who underwent cardiovascular bypass graft surgery (CABG) and were treated with pioglitazone or pioglitazone/simvastatin compared to the control group [79]. Dipeptidyl peptidase 4 (DPP-4) inhibitors are oral antidiabetic medications which regulate blood glucose levels by stimulating incretin production, inhibiting glucagon release and increasing insulin levels [80]. Sitagliptin is a DPP-4 inhibitor which has been investigated regarding its role in abnormal epicardial fat modulation [80]. Lima-Martínez et al. conducted an interventional study on 26 overweight and diabetic patients insufficiently treated with metformin [80]. The combination of sitagliptin/metformin at a dosage 50/1000 twice a day was registered to these subjects [80]. Epicardial fat was assessed before and after this medical intervention via ultrasound [80]. Significant reduction (from 9.98 ± 2.63 to 8.10 ± 2.11 mm, p = 0.001) in EAT was achieved after sitagliptin admission [80]. Glucagon-like peptide-1 (GLP-1) receptor agonists are injectable drugs which not only attenuate diabetic hyperglycaemia but also reduce obesity and major adverse cardiovascular events [81]. Epicardial adipocytes express GLP-1 receptor that has been shown to inhibit local adipogenesis by increasing free fatty acid oxidation to induce brown adipose tissue differentiation and to increase insulin sensitivity [75,82].

Current research has shown positive results in EAT thickness-reduction in diabetic patients who received either liraglutide, semaglutide, or dulaglutide [83,84]. Sodium-glucose co-transporter -2 (SGLT-2) inhibitors are novel hypoglycemic agents which block glucose reabsorption in the proximal kidney tubule and consequently result in glucosuria and serum glycemic control [85]. Administration of such agents leads to weight loss and decline in visceral adiposity [85]. Particularly, SGLT-2 inhibitors-induced hypoglycemia promotes lipolysis, free fatty acid oxidation and improved visceral lipid and glucose metabolism [86]. Noticeable reduction of EAT volume, serum inflammatory cytokines and increase in adiponectin levels were identified in 40 diabetic individuals with CAD after the administration of dapagliflozin for six months, indicating a positive role of SGLT-2 in EAT function and overall metabolic profile [87]. Similar results were also noticed after the administration of empagliflozin [88]. Insulin restoring treatment is also associated to EAT thickness reduction [89]. In 2016, Elisha et al. denoted a clear decline in epicardial fat composition in diabetes mellitus 2 patients who were treated with either detemir or glargine [89]. However, the insulin effect was more prominent in the detemir group [89].

## Conclusions

Nowadays, special attention is paid to the complex physiology and pathophysiology properties of epicardial fat. Epicardial adipose tissue is now considered a determining cardiometabolic risk factor for cardiovascular diseases, especially CAD. Multiple studies have been conducted to investigate the plausible pathophysiology pathways regarding abnormal EAT and coronary atherosclerosis progression. Elevated secretion of inflammatory molecules and significant reduction of anti-inflammatory ones, the elevation of immunity cells reaction, increased levels of reactive oxygen species, lipotoxicity and glucotoxicity have been proposed as the main etiopathogenic routes. Taking all these pleiotropic effects of epicardial fat into account, accurate measurement of this fat unit seems to be essential. Imaging techniques, such as conventional echocardiography, CT, and MRI should be easily accessible in everyday clinical practice for the measurement of EAT. To the best of our knowledge, both lifestyle and pharmacological interventions could modulate abnormal EAT volume and thickness, restore its cardioprotective effects and eliminate coronary atherosclerosis progression. A healthy lifestyle and medical treatment with either traditional or novel antidiabetic and lipid-lowering agents have shown encouraging results. More clinical investigations should be conducted to better understand the exact pathophysiology mechanisms of EAT and determine whether the pharmacological alteration of its mass could efficiently prevent CAD.

## References

[1] Sanchis-Gomar F, Perez-Quilis C, Leischik R, Lucia A (2016). Epidemiology of coronary heart disease and acute coronary syndrome. Ann Transl Med.

[2] (2020). Correction to: heart disease and stroke statistics-2019 update: a report from the American Heart Association. Circulation.

[3] Malakar AK, Choudhury D, Halder B, Paul P, Uddin A, Chakraborty S (2019). A review on coronary artery disease, its risk factors, and therapeutics. J Cell Physiol.

[4] Agrawal H, Choy HK, Liu J, Auyoung M, Albert MA (2020). Coronary artery disease. Arterioscler Thromb Vasc Biol.

[5] Wang Y, Li J, Zheng X (2018). Risk factors associated with major cardiovascular events 1 year after acute myocardial infarction. JAMA Netw Open.

[6] Gadde KM, Heymsfield SB (2021). Targeting visceral adiposity with pharmacotherapy. Lancet Diabetes Endocrinol.

[7] Koenen M, Hill MA, Cohen P, Sowers JR (2021). Obesity, adipose tissue and vascular dysfunction. Circ Res.

[8] Patel VB, Shah S, Verma S, Oudit GY (2017). Epicardial adipose tissue as a metabolic transducer: role in heart failure and coronary artery disease. Heart Fail Rev.

[9] Antonopoulos AS, Antoniades C (2017). The role of epicardial adipose tissue in cardiac biology: classic concepts and emerging roles. J Physiol.

[10] Bertaso AG, Bertol D, Duncan BB, Foppa M (2013). Epicardial fat: definition, measurements and systematic review of main outcomes. Arq Bras Cardiol.

[11] Iacobellis G (2016). Epicardial fat: a new cardiovascular therapeutic target. Curr Opin Pharmacol.

[12] Verma B, Katyal D, Patel A, Singh VR, Kumar S (2019). Relation of systolic and diastolic epicardial adipose tissue thickness with presence and severity of coronary artery disease (The EAT CAD study). J Family Med Prim Care.

[13] Ansaldo AM, Montecucco F, Sahebkar A, Dallegri F, Carbone F (2019). Epicardial adipose tissue and cardiovascular diseases. Int J Cardiol.

[14] Villasante Fricke AC, Iacobellis G (2019). Epicardial adipose tissue: clinical biomarker of cardio-metabolic risk. Int J Mol Sci.

[15] Mazurek T, Zhang L, Zalewski A (2003). Human epicardial adipose tissue is a source of inflammatory mediators. Circulation.

[16] Noyes AM, Dua K, Devadoss R, Chhabra L (2014). Cardiac adipose tissue and its relationship to diabetes mellitus and cardiovascular disease. World J Diabetes.

[17] Iacobellis G (2015). Local and systemic effects of the multifaceted epicardial adipose tissue depot. Nat Rev Endocrinol.

[18] Monti CB, Codari M, De Cecco CN, Secchi F, Sardanelli F, Stillman AE (2020). Novel imaging biomarkers: epicardial adipose tissue evaluation. Br J Radiol.

[19] Conte M, Petraglia L, Poggio P (2022). Inflammation and cardiovascular diseases in the elderly: the role of epicardial adipose tissue. Front Med (Lausanne).

[20] Willens HJ, Gómez-Marín O, Chirinos JA, Goldberg R, Lowery MH, Iacobellis G (2008). Comparison of epicardial and pericardial fat thickness assessed by echocardiography in African American and non-Hispanic White men: a pilot study. Ethn Dis.

[21] Venuraju SM, Lahiri A, Jeevarethinam A, Rakhit RD, Shah PK, Nilsson J (2021). Association of epicardial fat volume with the extent of coronary atherosclerosis and cardiovascular adverse events in asymptomatic patients with diabetes. Angiology.

[22] Christensen RH, von Scholten BJ, Lehrskov LL, Rossing P, Jørgensen PG (2020). Epicardial adipose tissue: an emerging biomarker of cardiovascular complications in type 2 diabetes?. Ther Adv Endocrinol Metab.

[23] Gormez S, Erdim R, Akan G (2020). Relationships between visceral/subcutaneous adipose tissue FABP4 expression and coronary atherosclerosis in patients with metabolic syndrome. Cardiovasc Pathol.

[24] Prati F, Arbustini E, Labellarte A (2003). Eccentric atherosclerotic plaques with positive remodelling have a pericardial distribution: a permissive role of epicardial fat? A three-dimensional intravascular ultrasound study of left anterior descending artery lesions. Eur Heart J.

[25] Iacobellis G (2021). Aging effects on epicardial adipose tissue. Frontiers.

[26] Fainberg HP, Birtwistle M, Alagal R (2018). Transcriptional analysis of adipose tissue during development reveals depot-specific responsiveness to maternal dietary supplementation. Sci Rep.

[27] Sacks HS, Fain JN, Holman B (2009). Uncoupling protein-1 and related messenger ribonucleic acids in human epicardial and other adipose tissues: epicardial fat functioning as brown fat. J Clin Endocrinol Metab.

[28] Sacks HS, Fain JN, Cheema P (2011). Inflammatory genes in epicardial fat contiguous with coronary atherosclerosis in the metabolic syndrome and type 2 diabetes: changes associated with pioglitazone. Diabetes Care.

[29] Packer M (2018). Epicardial adipose tissue may mediate deleterious effects of obesity and inflammation on the myocardium. J Am Coll Cardiol.

[30] Guglielmi V, Sbraccia P (2017). Epicardial adipose tissue: at the heart of the obesity complications. Acta Diabetol.

[31] Guglielmo M, Lin A, Dey D, Baggiano A, Fusini L, Muscogiuri G, Pontone G (2021). Epicardial fat and coronary artery disease: role of cardiac imaging. Atherosclerosis.

[32] Burgeiro A, Fonseca AC, Espinoza D, Carvalho L, Lourenço N, Antunes M, Carvalho E (2018). Proteostasis in epicardial versus subcutaneous adipose tissue in heart failure subjects with and without diabetes. Biochim Biophys Acta Mol Basis Dis.

[33] Wu S, Zou MH (2020). AMPK, mitochondrial function, and cardiovascular disease. Int J Mol Sci.

[34] Yamauchi T, Iwabu M, Okada-Iwabu M, Kadowaki T (2014). Adiponectin receptors: a review of their structure, function and how they work. Best Pract Res Clin Endocrinol Metab.

[35] Fang H, Judd RL (2018). Adiponectin regulation and function. Compr Physiol.

[36] Du Y, Ji Q, Cai L (2016). Association between omentin-1 expression in human epicardial adipose tissue and coronary atherosclerosis. Cardiovasc Diabetol.

[37] Li Y, Jiang C, Wang X, Zhang Y, Shibahara S, Takahashi K (2007). Adrenomedullin is a novel adipokine: adrenomedullin in adipocytes and adipose tissues. Peptides.

[38] Iacobellis G, di Gioia CR, Di Vito M (2009). Epicardial adipose tissue and intracoronary adrenomedullin levels in coronary artery disease. Horm Metab Res.

[39] Watanabe K, Watanabe R, Konii H (2016). Counteractive effects of omentin-1 against atherogenesis†. Cardiovasc Res.

[40] McAninch EA, Fonseca TL, Poggioli R (2015). Epicardial adipose tissue has a unique transcriptome modified in severe coronary artery disease. Obesity (Silver Spring).

[41] Henningsson M, Brundin M, Scheffel T, Edin C, Viola F, Carlhäll CJ (2020). Quantification of epicardial fat using 3D cine Dixon MRI. BMC Med Imaging.

[42] Iacobellis G, Assael F, Ribaudo MC, Zappaterreno A, Alessi G, Di Mario U, Leonetti F (2003). Epicardial fat from echocardiography: a new method for visceral adipose tissue prediction. Obes Res.

[43] Rado SD, Lorbeer R, Gatidis S (2019). MRI-based assessment and characterization of epicardial and paracardial fat depots in the context of impaired glucose metabolism and subclinical left-ventricular alterations. Br J Radiol.

[44] Wu Y, Zhang A, Hamilton DJ, Deng T (2017). Epicardial fat in the maintenance of cardiovascular health. Methodist Debakey Cardiovasc J.

[45] Sarin S, Wenger C, Marwaha A (2008). Clinical significance of epicardial fat measured using cardiac multislice computed tomography. Am J Cardiol.

[46] Lin A, Dey D, Wong DT, Nerlekar N (2019). Perivascular adipose tissue and coronary atherosclerosis: from biology to imaging phenotyping. Curr Atheroscler Rep.

[47] Shahzad R, Bos D, Metz C (2013). Automatic quantification of epicardial fat volume on non-enhanced cardiac CT scans using a multi-atlas segmentation approach. Med Phys.

[48] Neeland IJ, Ross R, Després JP (2019). Visceral and ectopic fat, atherosclerosis, and cardiometabolic disease: a position statement. Lancet Diabetes Endocrinol.

[49] Hirata Y, Kurobe H, Akaike M (2011). Enhanced inflammation in epicardial fat in patients with coronary artery disease. Int Heart J.

[50] Suganami T, Tanimoto-Koyama K, Nishida J (2007). Role of the Toll-like receptor 4/NF-kappaB pathway in saturated fatty acid-induced inflammatory changes in the interaction between adipocytes and macrophages. Arterioscler Thromb Vasc Biol.

[51] Baker AR, Harte AL, Howell N (2009). Epicardial adipose tissue as a source of nuclear factor-kappaB and c-Jun N-terminal kinase mediated inflammation in patients with coronary artery disease. J Clin Endocrinol Metab.

[52] Baker AR, Silva NF, Quinn DW (2006). Human epicardial adipose tissue expresses a pathogenic profile of adipocytokines in patients with cardiovascular disease. Cardiovasc Diabetol.

[53] McLaughlin T, Schnittger I, Nagy A (2021). Relationship between coronary atheroma, epicardial adipose tissue inflammation, and adipocyte differentiation across the human myocardial bridge. J Am Heart Assoc.

[54] Sinha SK, Thakur R, Jha MJ (2016). Epicardial adipose tissue thickness and its association with the presence and severity of coronary artery disease in clinical setting: a cross-sectional observational study. J Clin Med Res.

[55] Zhang T, Yang P, Li T, Gao J, Zhang Y (2019). Leptin expression in human epicardial adipose tissue is associated with local coronary atherosclerosis. Med Sci Monit.

[56] Montazerifar F, Bolouri A, Paghalea RS, Mahani MK, Karajibani M (2016). Obesity, serum resistin and leptin levels linked to coronary artery disease. Arq Bras Cardiol.

[57] Sardu C, D'Onofrio N, Torella M (2019). Pericoronary fat inflammation and major adverse cardiac events (MACE) in prediabetic patients with acute myocardial infarction: effects of metformin. Cardiovasc Diabetol.

[58] Elsanhoury A, Nelki V, Kelle S, Van Linthout S, Tschöpe C (2021). Epicardial fat expansion in diabetic and obese patients with heart failure and preserved ejection fraction-a specific HFpEF phenotype. Front Cardiovasc Med.

[59] Demir B, Demir E, Acıksarı G (2014). The association between the epicardial adipose tissue thickness and oxidative stress parameters in isolated metabolic syndrome patients: a multimarker approach. Int J Endocrinol.

[60] Camarena V, Sant D, Mohseni M, Salerno T, Zaleski ML, Wang G, Iacobellis G (2017). Novel atherogenic pathways from the differential transcriptome analysis of diabetic epicardial adipose tissue. Nutr Metab Cardiovasc Dis.

[61] Vyas V, Blythe H, Wood EG (2021). Obesity and diabetes are major risk factors for epicardial adipose tissue inflammation. JCI Insight.

[62] Salgado-Somoza A, Teijeira-Fernández E, Rubio J, Couso E, González-Juanatey JR, Eiras S (2012). Coronary artery disease is associated with higher epicardial retinol-binding protein 4 (RBP4) and lower glucose transporter (GLUT) 4 levels in epicardial and subcutaneous adipose tissue. Clin Endocrinol (Oxf).

[63] Erkan AF, Tanindi A, Kocaman SA, Ugurlu M, Tore HF (2016). Epicardial adipose tissue thickness is an independent predictor of critical and complex coronary artery disease by Gensini and syntax scores. Tex Heart Inst J.

[64] Gorter PM, de Vos AM, van der Graaf Y (2008). Relation of epicardial and pericoronary fat to coronary atherosclerosis and coronary artery calcium in patients undergoing coronary angiography. Am J Cardiol.

[65] Cosson E, Nguyen MT, Rezgani I (2021). Epicardial adipose tissue volume and coronary calcification among people living with diabetes: a cross-sectional study. Cardiovasc Diabetol.

[66] Ito T, Suzuki Y, Ehara M (2013). Impact of epicardial fat volume on coronary artery disease in symptomatic patients with a zero calcium score. Int J Cardiol.

[67] de Vos AM, Prokop M, Roos CJ (2008). Peri-coronary epicardial adipose tissue is related to cardiovascular risk factors and coronary artery calcification in post-menopausal women. Eur Heart J.

[68] Iacobellis G, Singh N, Wharton S, Sharma AM (2008). Substantial changes in epicardial fat thickness after weight loss in severely obese subjects. Obesity (Silver Spring).

[69] Konwerski M, Postuła M, Barczuk-Falęcka M (2021). Epicardial adipose tissue and cardiovascular risk assessment in ultra-marathon runners: a pilot study. Int J Environ Res Public Health.

[70] Saco-Ledo G, Valenzuela PL, Castillo-García A, Arenas J, León-Sanz M, Ruilope LM, Lucia A (2021). Physical exercise and epicardial adipose tissue: a systematic review and meta-analysis of randomized controlled trials. Obes Rev.

[71] Altin C, Erol V, Aydin E (2018). Impact of weight loss on epicardial fat and carotid intima-media thickness after laparoscopic sleeve gastrectomy: a prospective study. Nutr Metab Cardiovasc Dis.

[72] Kang J, Kim YC, Park JJ (2018). Increased epicardial adipose tissue thickness is a predictor of new-onset diabetes mellitus in patients with coronary artery disease treated with high-intensity statins. Cardiovasc Diabetol.

[73] Oesterle A, Laufs U, Liao JK (2017). Pleiotropic effects of statins on the cardiovascular system. Circ Res.

[74] Raggi P, Gadiyaram V, Zhang C, Chen Z, Lopaschuk G, Stillman AE (2019). Statins reduce epicardial adipose tissue attenuation independent of lipid lowering: a potential pleiotropic effect. J Am Heart Assoc.

[75] Dozio E, Ruscica M, Vianello E (2020). PCSK9 expression in epicardial adipose tissue: molecular association with local tissue inflammation. Mediators Inflamm.

[76] Rivas Galvez RE, Morales Portano JD, Trujillo Cortes R, Gomez Alvarez EB, Sanchez Cubias SM, Zelaya SM (2020). Reduction of epicardial adipose tissue thickness with PCSK9inhibitors. European Heart Journal.

[77] Greulich S, Maxhera B, Vandenplas G (2012). Secretory products from epicardial adipose tissue of patients with type 2 diabetes mellitus induce cardiomyocyte dysfunction. Circulation.

[78] Ziyrek M, Kahraman S, Ozdemir E, Dogan A (2019). Metformin monotherapy significantly decreases epicardial adipose tissue thickness in newly diagnosed type 2 diabetes patients. Rev Port Cardiol (Engl Ed).

[79] Grosso AF, de Oliveira SF, Higuchi Mde L, Favarato D, Dallan LA, da Luz PL (2014). Synergistic anti-inflammatory effect: simvastatin and pioglitazone reduce inflammatory markers of plasma and epicardial adipose tissue of coronary patients with metabolic syndrome. Diabetol Metab Syndr.

[80] Lima-Martínez MM, Paoli M, Rodney M, Balladares N, Contreras M, D'Marco L, Iacobellis G (2016). Effect of sitagliptin on epicardial fat thickness in subjects with type 2 diabetes and obesity: a pilot study. Endocrine.

[81] Dozio E, Vianello E, Malavazos AE, Tacchini L, Schmitz G, Iacobellis G, Corsi Romanelli MM (2019). Epicardial adipose tissue GLP-1 receptor is associated with genes involved in fatty acid oxidation and white-to-brown fat differentiation: a target to modulate cardiovascular risk?. Int J Cardiol.

[82] Morano S, Romagnoli E, Filardi T (2015). Short-term effects of glucagon-like peptide 1 (GLP-1) receptor agonists on fat distribution in patients with type 2 diabetes mellitus: an ultrasonography study. Acta Diabetol.

[83] Iacobellis G, Villasante Fricke AC (2020). Effects of semaglutide versus dulaglutide on epicardial fat thickness in subjects with type 2 diabetes and obesity. J Endocr Soc.

[84] Iacobellis G, Mohseni M, Bianco SD, Banga PK (2017). Liraglutide causes large and rapid epicardial fat reduction. Obesity (Silver Spring).

[85] Vallon V, Verma S (2021). Effects of SGLT2 inhibitors on kidney and cardiovascular function. Annu Rev Physiol.

[86] Carbone S, O'Keefe JH, Lavie CJ (2020). SGLT2 inhibition, visceral adiposity, weight, and type 2 diabetes mellitus. Obesity (Silver Spring).

[87] Sato T, Aizawa Y, Yuasa S (2018). The effect of dapagliflozin treatment on epicardial adipose tissue volume. Cardiovasc Diabetol.

[88] Anker SD, Butler J, Filippatos GS (2019). Evaluation of the effects of sodium-glucose co-transporter 2 inhibition with empagliflozin on morbidity and mortality in patients with chronic heart failure and a preserved ejection fraction: rationale for and design of the EMPEROR-Preserved Trial. Eur J Heart Fail.

[89] Elisha B, Azar M, Taleb N, Bernard S, Iacobellis G, Rabasa-Lhoret R (2016). Body composition and epicardial fat in type 2 diabetes patients following insulin detemir versus insulin glargine initiation. Horm Metab Res.

